# Investigating the Effectiveness of a Family Intervention after Acquired Brain or Spinal Cord Injury: A Randomized Controlled Trial

**DOI:** 10.3390/jcm12093214

**Published:** 2023-04-29

**Authors:** Pernille Langer Soendergaard, Juan Carlos Arango-Lasprilla, Mia Moth Wolffbrandt, Frederik Lehman Dornonville de la Cour, Fin Biering-Sørensen, Anne Norup

**Affiliations:** 1 Neurorehabilitation Research and Knowledge Centre, Rigshospitalet, 2600 Glostrup, Denmark; mia.moth.wolffbrandt@regionh.dk (M.M.W.); frederik.dornonville.de.la.cour@regionh.dk (F.L.D.d.l.C.); 2 Department of Psychology, University of Southern Denmark, 5230 Odense, Denmark; 3 Neurorehabilitation-CPH, City of Copenhagen, 2900 Hellerup, Denmark; 4 Department of Psychology, Virginia Commonwealth University, Richmond, VA 23284, USA; jcalasprilla@gmail.com; 5 Department of Neuroscience, University of Copenhagen, 2200 Copenhagen, Denmark; 6 The Elsass Foundation, 2920 Charlottenlund, Denmark; 7 Department of Clinical Medicine, University of Copenhagen, 2200 Copenhagen, Denmark; fin.biering-soerensen@regionh.dk; 8 Department of Brain and Spinal Cord Injuries, Rigshospitalet, Copenhagen University Hospital, 2600 Glostrup, Denmark

**Keywords:** acquired brain injury, spinal cord injury, individual with injury, caregiver, family intervention, quality of life, caregiver burden, randomized controlled trial

## Abstract

(1) Background: Acquired brain injury (ABI) or spinal cord injury (SCI) constitutes a severe life change for the entire family, often resulting in decreased quality of life (QoL) and increased caregiver burden. The objective of this study was to investigate the effectiveness of a family intervention in individuals with ABI or SCI and in their family members. (2) Methods: An RCT of a family intervention group (FIG) vs. a psychoeducational group (PEG) (ratio 1:1) was performed. The FIG received an eight-week manual-based family intervention, and the PEG received one psychoeducational session. Self-reported questionnaires on QoL with the Mental Component Summary (MCS) and on caregiver burden with the Caregiver Burden Scale (CBS) were the primary outcomes. The data analysis involved linear mixed-effects regression models. (3) Results: In total, 74 participants were allocated randomly to the FIG and 84 were allocated randomly to the PEG. The FIG had significantly larger improvements on the MCS and significantly larger reductions on the CBS at the two-month follow-up than participants in the PEG (mean differences of 5.64 points on the MCS and −0.26 points on the CBS). At the eight-month follow-up, the between-group difference remained significant (mean difference of 4.59 points) on the MCS, whereas that on the CBS was borderline significant (mean change of −0.14 points). (4) Conclusions: Family intervention was superior to psychoeducation, with larger improvements in QoL and larger reductions in caregiver burden.

## 1. Introduction

Acquired brain injury (ABI) or spinal cord injury (SCI) present significant challenges not only for the individual who sustains the injury but also for the surrounding family, as they all must adapt to the suddenly changed life situation [1,2,3,4,5,6]. In Denmark, it is estimated that approximately 230,000 individuals are living with the consequences of ABI and, similarly, 3000 individuals are living with the consequences of SCI [7,8,9]. Despite the differences in etiology, both ABI and SCI are complex and life-threatening conditions that present long-term challenges that neither the individual with the injury nor the family members have been prepared for or have prior experiences with.

An injury can affect family functioning in the long-term, with increased emotional burden, high frequencies of symptoms of anxiety and depression, and lower quality of life (QoL) reported up to 20 years after the injury [1,5,10,11,12,13,14,15,16,17]. Furthermore, despite the treatment provided from a formal health care system, informal care relies on family members, which can pose a significant caregiver burden [15,18,19,20,21,22], causing an imbalance in the family system [2,23] and a threat to the family relationship [17,24,25,26]. Increased levels of burden on family members can furthermore have an impact on their level of psychological distress, which can affect their ability to provide care. Consequently, the outcome of the individual with the injury may be negatively affected [2,15].

Therefore, to meet the complex changes within the family and to improve the overall family function, interventions addressing the entire family and targeting the family as a system may be a solution [27,28]. However, the paradigm of supporting the family after an injury has long been based on psychoeducation, skill-building, problem-solving strategies [29,30,31,32,33,34,35], emotional support, psychosocial interventions, or self-care. These interventions have primarily targeted the primary family member of the family [20,31,36], often a spouse or a partner [4,19,37], and the individual with the injury separately [4,15,27,28]. However, it has not been investigated longitudinally how such interventions affect all members of the family as only few cross-sectional studies have been carried out [38,39], and only few studies have been conducted on interventions for the entire family together [5,27,32,40].

Consequently, few family-centered interventions after ABI and almost none after SCI have been developed. Furthermore, the effectiveness of such interventions are sparsely investigated [4,15,27,32,41]; therefore, evidence-based knowledge and controlled studies on family systems and family-centered interventions are needed [27,28,42].

Therefore, the purpose of this study was to investigate the effectiveness of an eight-week manual-based family intervention in individuals with ABI or SCI and in their family members to improve mental-health-related QoL and to reduce caregiver burden.

It was hypothesized that participants receiving the family intervention experienced significantly larger improvements in mental-health-related QoL and significantly larger reductions in caregiver burden compared with participants receiving one psychoeducational session. 

Given that an injury influences and changes the family system, the longitudinal investigation presented in the present study on the effectiveness of this structured approach to facilitate change across the family system is important for gaining knowledge on how to support the individual with injury and the family in the best possible way in their changed life situation [28,42].

## 2. Materials and Methods

### 2.1. Study Design

The study was a two-arm randomized controlled trial (RCT) conducted in Denmark. Individuals with ABI or SCI were randomly assigned to a family intervention group (FIG) or a psychoeducational group (PEG) together with their family members. All participants provided written informed consent in concordance with the Helsinki Declaration [43]. The study was reported to the Danish Data Protection Agency (journal no. P-2021-603) and to the Committees on Health Research Ethics on the Capital Region of Denmark (journal no. H-1801 4858). The RCT was registered on 24 January 2019 at ClinicalTrials.gov, identifier: NCT03814876, where the protocol, 2018_0004, Family Intervention Following Traumatic Injury, is accessible. The study protocol was published in 2019 [44], and this study was reported according to the Consolidated Standards of Reporting Randomized Trials of Psychosocial Interventions (CONSORT-SPI) [45].

### 2.2. Participants

Individuals with traumatic injuries and their family members were enrolled from October 2018 to June 2021, and in addition, inclusion of individuals with non-traumatic injuries and their family members occurred between November 2019 and June 2021. Consequently, the ABI population and the SCI population included both traumatic and non-traumatic injuries. The traumatic brain injuries (TBI) were caused by either a blow to the head or a penetrating injury, whereas the non-traumatic brain injuries (NTBI) were caused by, e.g., cerebrovascular diseases, ischemic or hemorrhagic stroke, or infections [46]. The traumatic spinal cord injuries (tSCI) were caused by, e.g., falls, traffic accidents, sport accidents, or violence. In addition, the non-traumatic injuries (ntSCI) were caused by, e.g., infections, or degeneration and diseases with spinal stenosis or prolapsed discs [47,48]. 

Individuals with ABI or SCI were primarily recruited from two highly specialized neurorehabilitation departments in the Eastern part of Denmark (Department of Brain Injuries, Rigshospitalet, and Department of Spinal Cord Injuries, Rigshospitalet) between six months and two years after discharge. Research assistants screened the individuals with injuries and their family members for eligibility. All individuals with injuries participated with at least one family member (spouse, partner, adult children, parents, or siblings) who they described as actively involved in their life. All participants were ≥18 years old at the time of inclusion, able to understand and speak Danish, and cognitively able to participate in the study (Rancho Los Amigos Scale ≥ 7, Mini Mental State Examination score ≥23 [49], and no severe aphasia at time of inclusion for individuals with ABI). Participants were excluded, if they were previously diagnosed with another neurologic or psychiatric disorder, had experienced violence in their family, or were struggling with substance abuse at the time of inclusion. 

### 2.3. Intervention

Both the family intervention and the psychoeducational session were a supplement to treatment-as-usual (TAU), as in Denmark, individuals with injuries and family members are not systematically offered any family-involving interventions together either during or after rehabilitation.

Initially, the family intervention and psychoeducational session were given in person at a highly specialized hospital in Copenhagen; however, from March 2020, the study had to undergo an adaptation because of the COVID-19 pandemic. Consequently, both the family intervention and psychoeducational session were given via videoconferencing during lockdowns in Denmark. 

#### 2.3.1. Family Intervention

Participants allocated to the FIG received an eight-week manual-based family intervention entitled Traumatic Brain Injury (TBI) / Spinal Cord Injury (SCI) Family Intervention [27] (referred to as ‘family intervention’) including all members of the family ≥ 18 years old agreeing to participate. The family intervention was theoretically founded with evidence-based strategies to improve well-being and psychological functioning for the individual with the injury and the family members [27]. The family intervention relied on strategies from rehabilitation psychology, cognitive behavioral therapy, and marriage and family therapy. The content of the 90 min eight-weekly sessions included different topics: making meaning and sharing experiences of the injury, shifting to a positive focus, managing emotions, communicating effectively, finding new solutions, and boundary making [27,40,44]. The family intervention was delivered to each family separately according to the manual by three different trained neuropsychologists with experience in neurorehabilitation. The content of the structured sessions comprised practical and theoretical components. Each session followed the same structure, starting with a discussion of a quotation relevant to the topic of the session, followed by a review of progress on practice tasks and then by background information about the topic and practicing new techniques. Between-session tasks were distributed to be completed between each session [27,40,44]. An overview of each session is outlined in Table 1 [27,44]. 

#### 2.3.2. Psychoeducation

Participants allocated to the PEG received a two-hour psychoeducational session on the consequences of an injury. The psychoeducation was administered to groups of families or individual families by an experienced neuropsychologist. The content of the psychoeducation was theoretically based information concerning the consequences of an injury, and how an injury can affect both the individual with the injury and the family members in the short and long terms, including not only the physical, cognitive, social, and mental consequences for the individual with the injury but also the reactions of the entire family including risk for higher distress, social isolation, burden, unmet needs, and lower QoL. The content was delivered orally, accompanied by written information to ensure consistency to all participants. However, at the end of the sessions, the participants were asked to elaborate on the topics, either within the family or between families, and therefore, the content could differ according to the families’ experiences and eagerness to share their thoughts.

### 2.4. Outcomes

The effectiveness of the family intervention was investigated on self-reported questionnaires completed by all participants at baseline, at the two-month follow-up, and at the eight-month follow-up. Questionnaires were selected based on empirical evidence on the consequences of an injury and on the constructs targeted in the family intervention [18,27]. 

#### 2.4.1. Primary Outcome Measures

The primary outcomes included the following:

Mental-health-related QoL was measured with the Mental Component Summary (MCS), which is a sum score on the mental health subscales (Vitality, Social Functioning, Role Emotional, and Mental Health) of the 36-item Short-Form Health Survey (SF-36v2) [50,51]. Scores are from 0 to 100, with higher scores indicating better health status. It was completed by all participants.

Caregiver burden was measured with the Caregiver Burden Scale (CBS), which is a multidimensional scale assessing the perceived subjective burden within general strain, isolation, disappointment, emotional involvement, and environment. Scores are from 1 to 4, with index scores of 1.00 to 1.99 indicating low burden, 2.00 to 2.99 indicating moderate burden, and 3.00 to 4.00 indicating high burden [52]. It was completed by all family members of the individual with the injury. 

#### 2.4.2. Secondary Outcome Measures

The secondary outcomes completed by all participants included the following:

Symptoms of anxiety were measured with the General Anxiety Disorder-7 (GAD-7). Total scores ranged from 0 to 21 on the seven items, with higher scores indicating more severe symptoms of anxiety (scores of 0–5, 6–10, 11–15, and 15–21 as cut points for mild, moderate, moderately severe, and severe anxiety, respectively) [53].

Symptoms of depression were measured with the Patient Health Questionnaire-9 (PHQ-9). Total scores ranged from 0 to 27 on the nine items, with higher scores indicating more severe symptoms of depression (scores of 0–4, 5–9, 10–14, 15–19, and 20–27 as cut points for minimal, mild, moderate, moderate severe, and severe depression, respectively) [54].

Cohesion and family flexibility were measured with the Circumplex Ratio Score from The Family Adaptability and Cohesion Evaluation Scale fourth edition (FACES-IV). Scores ranged from 0 to 10, with higher scores indicating better status (a score of ≥ 1 indicates balance in the levels of cohesion and flexibility in the family system) [55]. Additionally, level of communication and satisfaction with the family were assessed with the FACES-IV using the 10-item Family Communication Scale (FCS) and the 10-item Family Satisfaction Scale (FSS), respectively, with higher scores indicating better communication and higher satisfaction (ranging from 10 to 99) [55]. 

### 2.5. Sample Size

Preliminary power calculations were carried out based on the MCS from SF-36v2 and the CBS to detect the required sample sizes [44]. 

For the calculation on the MCS, a Norwegian study using SF-36 was used [56]. In the study, a mean score of 43.8 (SD 12.5) was reported for individuals with moderate to severe TBI, with a difference of 5.00 points between groups. Based on a significance level of 5% and a power of 80%, the predicted sample that needed to be recruited was 182 participants, with 91 participants allocated to each arm. 

For the calculation on the CBS, a Norwegian study using CBS was used [57]. In the study, a reduction of 0.40 points on the CBS for family members of individuals with TBI represented a moderate effect size. Based on a significance level of 5% and a power of 80%, the predicted sample that needed to be recruited was 126 family members, with 63 family members allocated to each arm [40]. 

### 2.6. Randomisation and Blinding

Individuals with ABI or SCI were randomly assigned to each arm together with their family members, with an allocation ratio 1:1. Participants were randomized at the end of session one by research assistants using the online software application Sealed Envelope [58]. The envelope was opened in the presence of the family. To ensure allocation concealment, computer-generated block sequences with randomization block sizes of 22 were produced. Blinding of the group allocation was not feasible for the participants or the neuropsychologists facilitating the family intervention or the psychoeducational session. Group allocation was masked during data analysis and interpretation of the results for all authors, and the masking was first revealed when the final analyses were completed. 

### 2.7. Analytical Methods

All analyses were conducted on an intention-to-treat basis, including all data available across time points. Between-group comparisons of change were analyzed for each outcome using linear mixed-effects regression models. Random effects included the two intercepts for individuals and families. Fixed effects included the main effects of group allocation (FIG and PEG) and time points (baseline, and two-month and eight-month follow-ups) and an interaction effect of group by time. In addition to crude analyses, the models were adjusted for the main effect of being an individual with injury or family member. The parameters were estimated using maximum likelihood estimation. The estimated marginal means were computed based on fitted models. The effect sizes were estimated using Cohen’s *d*. Assumptions of normality, homogeneity of variance, and linearity were examined. Analyses were conducted in R version 4.2.0 [59] using the *lme4* [60], *emmeans* [61], and *ggplot2* [62] packages. Missing data on MCS from SF-36v2 were imputed using the Missing Score Estimation (MSE) from Quality Metric Optum^®^ PRO CoRE Scoring Software. Imputation was possible if seven or more item scores were available, and the Mental Health scale was complete. On the GAD-7 and PHQ-9, missing data were imputed by the mean if one item score was missing on the GAD-7 and up to two item scores were missing on the PHQ-9, according to their respective manuals [53,54]. For the CBS and FACES-IV, listwise deletion was used to handle missing data [52,55]. 

## 3. Results

### 3.1. Participant Recruitment 

Between October 2018 and June 2021, a total of 157 participants were recruited, corresponding to 73 individuals with ABI or SCI and 84 family members (73 families, including 53 families with ABI and 20 families with SCI). Except for five individuals with injuries, all individuals with ABI or SCI had received highly specialized neurorehabilitation at a hospital. Of the remaining five individuals, two were included through their home municipality and three contacted the research group. All participants met the inclusion criteria. 

Of the total group, 74 participants (individuals with injuries and family members) were randomized to the FIG and 83 were randomized to the PEG. In the FIG, 31 participants received the family intervention in the online format via videoconferencing, compared with 29 in the PEG. In the FIG, 14 participants discontinued the intervention and dropped out between session two and session six, and in the PEG, 14 participants did not participate in the psychoeducational session. At the two-month follow-up, 60 participants from the FIG completed the self-reported questionnaires, whereas 66 participants from the PEG did. At the eight-month follow-up, the number of attending participants was 57 from the FIG and 66 from the PEG. Data collection was completed in May 2022.

**Figure 1:**
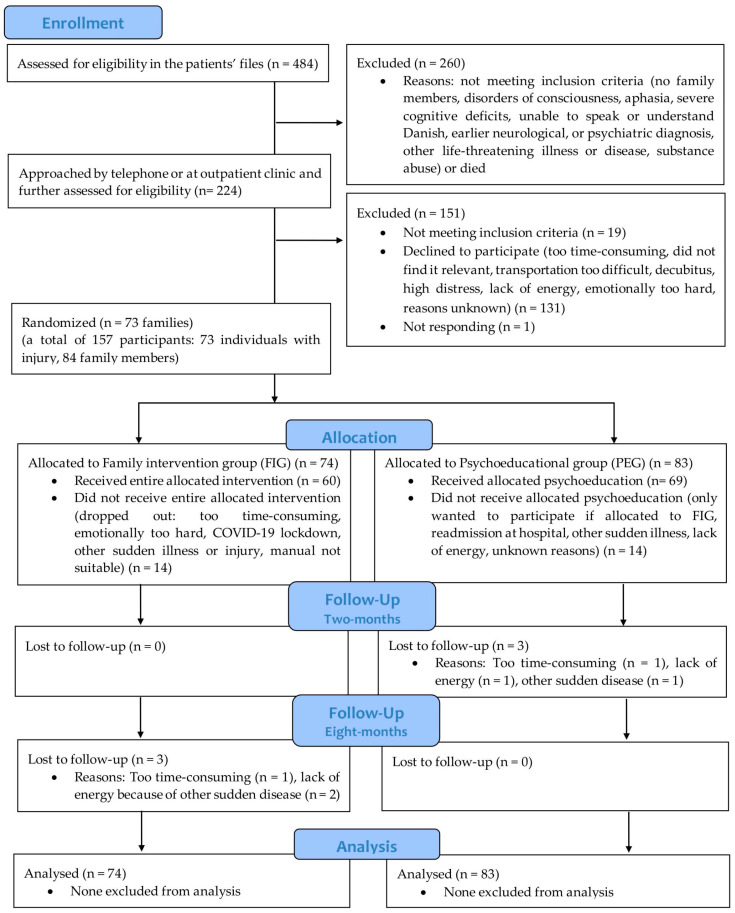
CONSORT 2010 flow diagram of recruitment of participants.

Figure 1 shows the flowchart of participant recruitment and a timeline of the follow-ups.

### 3.2. Baseline Characteristics

The demographic and injury characteristics according to intervention arm are depicted in Table 2.

Mean age in the FIG (53.40 years) was 3.05 years higher than that in the PEG, and sex distribution was 53% males in the FIG compared with 49% in the PEG. In both groups, kinship to the individual with the injury was mainly spouses or partners (71% in the FIG and 62% in the PEG), and most of the individuals with injuries were living together with the participating family member(s) (89% in the FIG and 82% in the PEG). In the FIG, 71% family members reported spending between one to five hours each day on helping, supervising, or caring for the individual with injury, correspondingly with 64% in the PEG. 

In both groups, injury was mainly caused by ABI (74% in the FIG and 71% in the PEG). All TBIs were classified as moderate to severe based on duration of post-traumatic amnesia (PTA) [63] (PTA for 51 days in the FIG and 39 days in the PEG), and most individuals with SCI were classified as grade D on the American Spinal Injury Association Impairment Scale, with incomplete impairments and motor functions preserved below the neurologic level (78% in the FIG and 73% in the PEG). The duration of neuro-intensive treatment followed by rehabilitation at a hospital also indicated the severity of the injuries [64] (Table 2). The groups did not differ significantly on any baseline characteristics or injury-related factors (all *p*’s > 0.05). 

### 3.3. Outcomes 

At baseline, groups did not differ significantly on primary or secondary outcomes (all *p*’s > 0.05).

#### 3.3.1. Primary Outcomes

From baseline to the two-month follow-up, participants allocated to the FIG improved by 4.96 points on the MCS, *p* < 0.001, d = 0.84, whereas participants allocated to the PEG decreased by −0.67 points, *p* = 0.725, d = −0.11, corresponding to a crude between-group difference in mean change of 5.64, *p* < 0.001, d = 0.96, in favor of the FIG. At the eight-month follow-up, the interaction effect remained significant in favor of the FIG by 4.59 points, *p* = 0.003, d = 0.78. 

On the CBS, the participants allocated to the FIG improved by −0.25 points, *p* < 0.001, d = −1.23, at the two-month follow-up, and −0.19 points, *p* = 0.002, d = −0.90, at the eight-month follow-up. For participants allocated to the PEG, no significant differences were found over time (two-month follow-up, *p* = 0.999; eight-month follow-up, *p* = 0.599). The between-group difference in crude mean change was in favor of the FIG, with −0.26 points, *p* < 0.001, d = −1.23, at the two-month follow-up, but at the eight-month follow-up, the difference between groups, with −0.14 points, *p* = 0.055, d = −0.70, was only borderline significant. 

**Figure 2:**
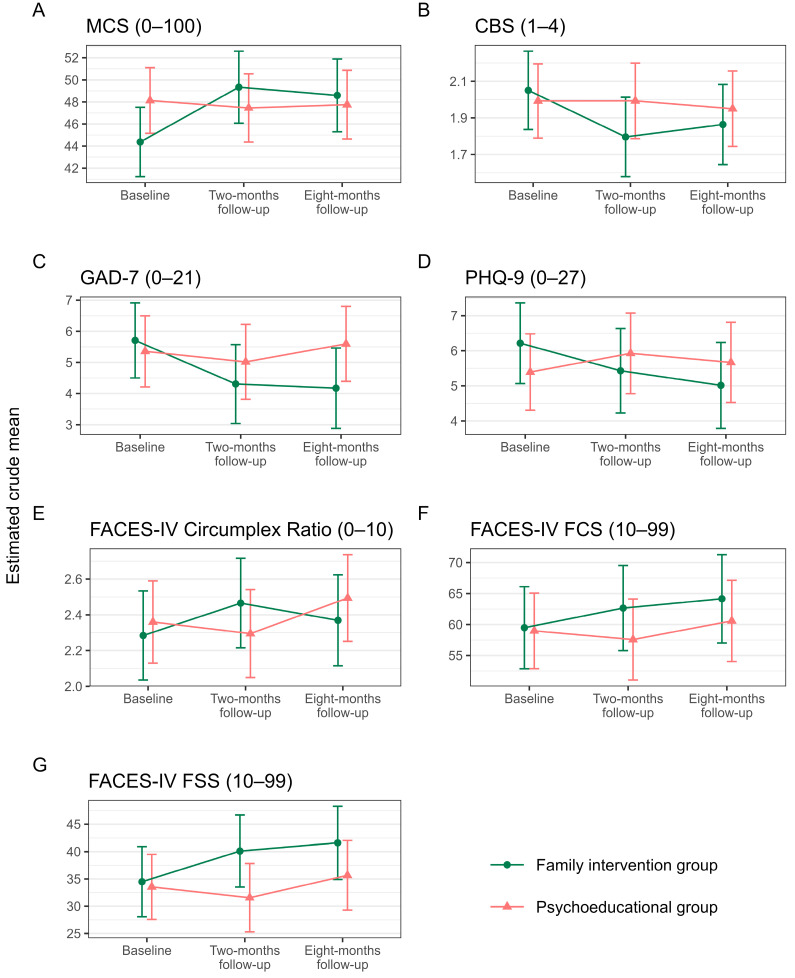
Estimated crude means on primary (**A**,**B**) and secondary (**C**–**G**) outcomes. Note: Error bars indicate 95% confidence intervals. Intervention was provided between baseline and two-month follow-up. MCS = Mental Component Summary (SF-36v2); CBS = Caregiver Burden Scale; GAD-7 = General Anxiety Disorder-7; PHQ-9 = Patient Health Questionnaire-9; FACES-IV = Family Adaptability and Cohesion Evaluation Scale IV; FCS = Family Communication Scale; FSS = Family Satisfaction Scale.

Detailed results of the primary outcomes are provided in the first rows of Table 3 and Figure 2A,B. Adjusting for the main effect of members in the family (individual with injury vs. family members) did not affect the results substantially for any outcome (Table 3). 

#### 3.3.2. Secondary Outcomes

The only significant within-group improvement in the secondary outcomes from baseline to the two-month follow-up were on the GAD-7 and were found in the FIG with an improvement of −1.40 points, *p* = 0.005, d = −0.56. Between-group differences in crude mean change from baseline to the two-month follow-up were found on the PHQ-9, with −1.32 points, *p* = 0.024, d = −0.58, and on the FSS, with 7.59 points, *p* = 0.031, d = 0.59, in favor of the FIG. No other outcomes were statistically significant. 

Compared with the PEG, only participants allocated to the FIG made significant larger within-group improvements from baseline to the eight-month follow-up on the GAD-7, with −1.54 points, *p* = 0.003, d = −0.61; on the PHQ-9, with −1.20 points, *p* = 0.012, d = −0.53; and on the FSS, with 7.11 points, *p* = 0.013, d = 0.55. The between-group differences in crude mean change from baseline to the eight-month follow-up was found in favor of the FIG on the GAD-7, with larger improvements of −1.77 points, *p* = 0.006, d = −0.71, and −1.48 points on the PHQ-9, *p* = 0.013, d = −0.65. No other outcomes were statistically significant. 

Detailed results on the five secondary outcomes are provided in the last rows of Table 3 and Figure 2C–G.

## 4. Discussion

The current study investigated the effectiveness of a family intervention developed for families living with the consequences of ABI or SCI to improve mental health, QoL, and family functioning among individuals with ABI and SCI and their family members. In consistency with our hypotheses, the results showed significant between-group differences, with the family intervention showing more benefits compared with psychoeducation on our primary outcome measures, including improvement in mental-health-related QoL at the two-month and eight-month follow-ups, and on reducing caregiver burden at the two-month follow-up, and borderline significance at the eight-month follow-up. Furthermore, for the secondary outcomes, the study showed a between-group benefit of the family intervention in addition to psychoeducation in reducing symptoms of depression and improving family satisfaction at the two-month follow-up, and in reducing symptoms of depression and symptoms of anxiety at the eight-month follow-up. 

Two other studies have investigated the effectiveness of the same family intervention and have found different results: a pilot study conducted in Latin America [27] and a RCT study conducted in Norway [40]. The results from the present RCT were consistent with the findings reported in the pilot study, which included individuals with SCI and their family members (n = 23 participants) [27]. Significant reductions in symptoms of depression, symptoms of anxiety, and caregiver burden were found in favor of the family intervention group at both the two-month and eight-month follow-ups compared with the waitlist control group. These results were comparable with our results using the same measures on anxiety and depression, but caregiver burden was measured using other assessments. However, a limitation of their study was a small sample size, which underpinned the need for further evaluation. This evaluation was carried out in the present RCT study, and our results confirm the results from the pilot study. 

The Norwegian RCT study did not find an effect of the family intervention when compared with a TAU group receiving one educational group session [40]. No significant between-group differences on mental-health-related QoL and caregiver burden were found; however, the Norwegian study did report improvements within the family intervention group. The Norwegian results differ from ours, and there are several differences between the populations included. In the Norwegian RCT study, families primarily facing consequences of mild TBI (mTBI) were included [40], which is in contrast to our study with moderate to severe injuries. The impact of mild injuries on the family might differ from the impact of more severe injuries regarding care activities and burden. In general, individuals with mild injuries often make a good recovery and improve in function over time [65]. The Norwegian study did report improvement in the TAU group. This could indicate that, even though they experienced persistent symptoms when they were included in the Norwegian study, the mTBI families might experience improvement naturally, and consequently, no extra benefit of the family intervention was reported. In our study, the PEG was stable in terms of the primary outcomes during the study period, and this was in contrast to the FIG, where changes were reported over time. 

The family intervention was developed for families living with the consequences of TBI and SCI and was designed for families facing serious injuries or illness [27]. The consequences of injuries, the need for support, and the general improvement in functioning over time might differ between the populations included in the three studies. Replications of the study in larger samples are needed to detect any differences in outcome between specific disease groups (ABI/TBI and SCI) and the severities of injury (mild and moderate/severe). The divergent findings between studies may suggest that the severity of injury might affect the relevance and appropriateness of the family intervention, and consequently, the severity of injury needs to be considered when offering a family intervention to families facing ABI or SCI. Furthermore, replications can contribute to investigations into any influences on outcome of participants characteristics (e.g., age of participants) and family-related factors (e.g., number of participating family members in the intervention and kinship). Future studies are also warranted to investigate the influence of the format of the family intervention on outcome (in person vs. videoconferencing). 

### Strengths and Limitations 

Strengths: First, a randomized design was adopted with a reasonable number of participants. Individuals with ABI or SCI were mainly recruited from two highly specialized rehabilitation departments, securing a uniform procedure for the inclusion. Second, the uptake area from the two departments covered the whole Eastern part of Denmark, which expanded the representativeness of the study. Third, the intervention was manual-based, ensuring the same information, psychoeducation, and techniques for each family, yet at the same time allowing for a flexible approach individualized for each family, as families chose specific challenges they were facing for the sessions. Fourth, the PEG received a psychoeducational session, which reduced the risk of bias compared with a TAU or a waitlist control group, e.g., the effects of participating in a clinical trial and being in contact with a healthcare professional. Fifth, loss to follow-up was low, which minimized the risk of bias.

Limitations: First, the generalizability of the study cannot be transferred to all families, as individuals with the most severe injuries were excluded (impaired consciousness, severe cognitive deficits, and aphasia), and only individuals willing to participate were included. There could be a selection bias, as the included participants might be families who either had the time and mental resources to participate or the opposite, where families with more impaired family functioning were eager to receive help and support. Second, the outcomes were only self-reported, which can be inflated by both placebo and nocebo effects. Due to the nature of the study, masking or blinding of the group allocation was not possible, which can have biased the participants’ responses on the self-reported questionnaires at the follow-up. Third, an incidental finding was that, despite the RCT design, the FIG and PEG differed on the MCS at baseline, with higher scores reported in the PEG, although non-significant (*p* = 0.080). Fourth, the intervention itself was carried out by three different neuropsychologists, and it cannot be ruled out that the efficacy of the family intervention might partly be due to the characteristics of the neuropsychologists. Fifth, the family intervention consisted of eight sessions with different components and techniques. Due to the design of the study, it was not possible to explore whether one component or technique was more important than others in achieving a change. However, several semi-structured interviews have been conducted in a concurrent, ongoing study, and these will be able to shed light on details regarding active ingredients of the intervention. 

Finally, the harms of the study need to be considered. A few participants from the family intervention withdrew during the intervention period as their experience with the content of the sessions was too emotional and painful to discuss with each other. A few others were forced to discontinue the intervention because of the COVID-19 lockdown. These families were invited to participate via videoconferencing instead of in person, but some declined. Others withdrew from the psychoeducational group, as they only participated hoping to be allocated to the family intervention group, and consequently, they felt disappointed and withdrew from the study. 

## 5. Conclusions

In conclusion, the family intervention was feasible and associated with a larger improvement in mental-health-related QoL and lower caregiver burden for participants allocated to the family intervention group. Consequently, the study contributed with novel knowledge on how to support the family in their changed life situation after ABI or SCI, suggesting that the family intervention was beneficial in reducing caregiver burden and improving mental-health-related QoL, as the family intervention was more effective than the psychoeducation. Additional data are needed to explore the active ingredients or components of the family intervention, and hopefully, semi-structured interviews of a concurrent study investigating the families’ experiences of the family intervention will provide insight into this. Focused replications of the study in other settings are needed, as well as those in larger multicenter trials, to detect, e.g., specific disease group outcomes and the severity of injury outcomes.

## Figures and Tables

**Table 1:** An overview of the sessions in the family intervention.

Session	Topic	Content
1	Introduction	The first session was a practical session, where the individual with the injury and the family members were introduced to the study. All participants completed the consent form and baseline questionnaires. Afterwards, the families were randomized to the family intervention group or the psychoeducational group.
2	Making meaning	The session consisted of psychoeducation on myths about an injury. The focus of the session was for the individual with the injury and the family members to share their experiences and thoughts on the consequences of an injury with each other.
3	Shifting focus	This session was about shifting focus, including the relationship among thoughts, feelings, and behavior, where the individual with the injury and family members were asked to think about the positive changes due to the injury, instead of what they were missing.
4	Managing emotions	In this session, the focus was on learning to identify signs of escalation in emotions. This session included techniques to manage these emotions, where the individual with the injury and the family members were asked to recognize their reactions in their body when they felt emotionally stressed.
5	Communicating effectively	This session consisted of numerous communication strategies, including what one should be aware of when communicating: I-statement strategy; talking–listening techniques; and communication-improving strategies, including communication danger signs.
6	Finding solutions	This session included problem-solving strategies, including focusing on solutions instead of the problem and formulating effectful goals (from problem talk to solution talk).
7	Boundary making	The focus of this session was on the importance of knowing the boundaries and roles in the family, including an awareness on healthier family dynamics and a focus on self-care activities.
8	Conclusion and farewell	Lastly, the individual with the injury and the family members were asked to reflect on the different topics of each session, what strategies they have used, and what they have benefitted most from. Thereafter, they completed the post intervention questionnaires before a short celebration, where the family received a diploma indicating that they, as a family, had completed the intervention together.

**Table 2:** Baseline characteristics of participants in each arm.

		FIG n = 74		PEG n = 83	
	n	Individuals with Injury n = 35 Family Members n = 39	n	Individuals with Injury n = 38 Family Members n = 45	*p* ^d^
Age, years (SD)	72	53.41 (16.90)	81	50.35 (14.47)	0.23
Sex, male, n (%)	74	39 (53)	83	41 (49)	0.68
Kinship to individual with Injury	39		45		0.50
Spouse/partner, n (%)		28 (71)		28 (62)	
Parent, n (%)		4 (10)		6 (13)	
Child, n (%)		3 (8)		8 (18)	
Sibling, n (%)		3 (8)		1 (2)	
Other, n (%)		1 (3)		2 (4)	
Length of relationship, years	67		66		0.07
<1, n (%)		1 (1)		5 (8)	
1–5, n (%)		5 (7)		8 (12)	
>5, n (%)		61 (91)		53 (80)	
Living with participating family member(s) ^a^, yes (%)	35	31 (89)	38	31 (82)	0.40
Level of education	73		83		0.72
Low, n (%)		27 (37)		33 (40)	
High ^b^, n (%)		46 (63)		50 (60)	
Employment status					
Pre-injury	72		83		0.47
Employed/student ^c^, n (%)		53 (74)		64 (77)	
Unemployed, n (%)		2 (3)		5 (6)	
Retired, n (%)		17 (24)		12 (14)	
On sick leave, n (%)		0		2 (2)	
Post-injury	72		81		0.32
Employed/student, n (%)		37 (51)		48 (59)	
Unemployed, n (%)		6 (8)		4 (5)	
Retired, n (%)		20 (28)		17 (21)	
On sick leave, n (%)		9 (13)		12 (15)	
Caring for individual with Injury each day, yes, n (%)	39	27 (69)	45	29 (64)	0.64
Hours per day	24		21		0.11
<1, n (%)		3 (13)		7 (33)	
1–5, n (%)		17 (71)		12 (57)	
>5, n (%)		4 (17)		2 (10)	
Previous psychological therapy, yes, n (%)	74	19 (26)	83	32 (39)	0.09
Receiving psychological therapy, yes, n (%)	73	7 (10)	82	11 (13)	0.46
Injury-related factors					
Previous rehabilitation, yes, n (%)	34	31 (91)	38	38 (100)	0.06
Neuro-intensive treatment, days, median (IQR)	32	25 (16–36)	36	22 (14–37)	0.72
Rehabilitation at hospital, days, median (IQR)	32	49 (32–65)	36	55 (42–76)	0.32
Receiving rehabilitation at inclusion, yes, n (%)	35	16 (46)	37	14 (38)	0.50
Acquired brain injury (n = 53)					
Cause of injury, TBI, n (%)	26	13 (50)	27	11 (41)	0.36
GCS at time of admission to rehabilitation at hospital, median (IQR)	21	14 (13–15)	25	14 (13–15)	0.81
PTA, days, median (IQR)	9	51 (26–52)	9	39 (26–58)	0.79
Spinal cord injury (n = 20)					
Cause of injury, tSCI, n (%)	9	7 (78)	11	6 (55)	0.29
Neurological level of injury	9		11		0.30
C2 to C4, n (%)		4 (44)		3 (27)	
C5 to Th1, n (%)		3 (33)		3 (27)	
Th2 to Th12, n (%)		1 (11)		3 (27)	
L1 to L5, n (%)		1 (11)		1 (9)	
S1 to S5, n (%)		0		1 (9)	
AIS grade	9		11		0.88
A, n (%)		2 (22)		2 (18)	
B, n (%)		0		0	
C, n (%)		0		1 (9)	
D, n (%)		7 (78)		8 (73)	
E, n (%)		0		0	

Note. ^a^ Living with at least one of the participating family members. ^b^ High level of education indicates a college or university degree. ^c^ Full-time or part-time employee/student. ^d^ ANOVA was used for continuous variables; the Wilcoxon rank-sum test was used for categorical variables. FIG, family intervention group; PEG, psychoeducational group; TBI, traumatic brain injury; GCS, Glasgow coma scale; PTA, post traumatic amnesia; tSCI, traumatic spinal cord injury; AIS, American Spinal Injury Association Impairment Scale.

**Table 3:** Outcomes at two-month and eight-month follow-up.

Measure	Total (n)		Baseline to Two-Month Follow-Up (95 % CI)				Baseline to Eight-Month Follow-Up (95 % CI)			
			Change in Estimated Mean		Between-Group Difference in Mean Change		Change in Estimated Means		Between-Group Difference in Mean Change	
	FIG	PEG	FIG	PEG	Crude	Adjusted ^a^	FIG	PEG	Crude	Adjusted ^a^
**Primary outcomes**										
MCS	189	211	4.96	–0.67	5.64	5.63	4.22	–0.37	4.59	4.6
			(2.55, 7.38) ***	(–2.95, 1.61)	(2.71, 8.56) ***	(2.71, 8.56) ***	(1.74, 6.69) ***	(–2.68, 1.93)	(1.61, 7.57) **	(1.62, 7.58) **
CBS ^b^	97	113	–0.25	0	–0.26	-	–0.19	–0.04	–0.14	-
			(–0.38, –0.13) ***	(–0.11, 0.11)	(–0.40, –0.11) ***		(–0.31, –0.06) **	(–0.15, 0.07)	(–0.29, 0.00)	
**Secondary outcomes**										
GAD-7	188	211	–1.40	–0.34	–1.06	–1.06	–1.54	0.24	–1.77	–1.77
			(–2.42, –0.38) **	(–1.31, 0.64)	(–2.30, 0.18)	(–2.30, 0.18)	(–2.60, –0.48) **	(–0.74, 1.21)	(–3.04, –0.51) **	(–3.04, –0.51) **
PHQ-9	187	209	–0.79	0.53	–1.32	–1.32	–1.20	0.28	–1.48	–1.48
			(–1.72, 0.15)	(–0.37, 1.44)	(–2.47, –0.17) *	(–2.47, –0.18) *	(–2.17, –0.23) *	(–0.62, 1.17)	(–2.64, –0.32) *	(–2.64, –0.32) *
FACES IV ^c^	150	156	0.18	–0.07	0.25	0.25	0.09	0.14	–0.05	–0.05
			(–0.02, 0.38)	(–0.27, 0.14)	(0.00, 0.50)	(0.00, 0.50)	(–0.12, 0.29)	(–0.07, 0.33)	(–0.30, 0.20)	(–0.30, 0.21)
FCS	166	188	3.17	–1.40	4.57	4.73	4.67	1.61	3.06	3.23
			(–3.28, 9.62)	(–7.61, 4.81)	(–3.32, 12.50)	(–3.14, 12.60)	(–2.07, 11.42)	(–4.60, 7.82)	(–5.01, 11.10)	(–4.82, 11.29)
FSS	167	181	5.62	–1.98	7.59	7.66	7.11	2.13	4.99	5
			(–0.02, 11.25)	(–7.42, 3.47)	(0.69, 14.50) *	(0.75, 14.60) *	(1.32, 12.90) *	(–3.49, 7.75)	(–2.12, 12.10)	(–2.11, 12.10)

Note. Means were estimated based on linear mixed models using maximum likelihood estimation in *R*. All models included time, group allocation, and the interaction of time and group allocation as fixed effects. Intercepts of individuals and family clusters were specified as random effects. FIG = family intervention group; PEG = psychoeducational group; CI = Confidence interval; MCS = Mental Component Summary (SF-36v2); CBS = Caregiver Burden Scale; GAD-7 = General Anxiety Disorder-7; PHQ-9 = Patient Health Questionnaire-9; FACES IV = Family Adaptability and Cohesion Evaluation Scale IV; FCS = Family Communication Scale; FSS = Family Satisfaction Scale. ^a^ Results were adjusted for the main effect of members in the family (individual with injury or family members). ^b^ Administered to family members only. ^c^ Total Circumplex Ratio was computed as outcome. * *p* < 0.05; ** *p* < 0.01; *** *p* < 0.001.

## Data Availability

The data presented in this study are available from the corresponding author upon request. The data are not publicly available due to privacy restriction and restriction stated by the Danish Data Protection Agency.
